# Strengthening Vaccine Safety Systems, Research, and Regional Collaboration in Africa: A Call to Action

**DOI:** 10.3390/vaccines13060661

**Published:** 2025-06-19

**Authors:** Beckie N. Tagbo, Chioma S. Ejekam, Winfred Oppong-Amoako, Tene Marceline Yameogo, Afework Mitiku, Dorothy O. Esangbedo, Nelisiwe Khuzwayo, Gugu Mahlangu, Samia M. Badar, Edinam A. Agbenu, Rhanda M. Adechina, Kwasi A. Nyarko

**Affiliations:** 1 Africa Advisory Committee on Vaccine Safety, WHO Regional Office for Africa, Brazzaville P.O. Box 06, Congo; tagbobeckie@gmail.com (B.N.T.); woamoako@gmail.com (W.O.-A.); teneline@gmail.com (T.M.Y.); afeworkassefa@gmail.com (A.M.); derion2@gmail.com (D.O.E.); khuzwayone@ukzn.ac.za (N.K.); gnmahlangu@gmail.com (G.M.); 2 Institute of Molecular Medicine & Infectious Disease, Department of Paediatrics, College of Medicine, University of Nigeria, Ituku-Ozalla Campus, Enugu 402109, Nigeria; 3 Vaccine Preventable Disease, Universal Health Coverage, Communicable and Non-Communicable Disease Cluster, WHO Regional Office for Africa, Brazzaville P.O. Box 06, Congo; mohamedsam@who.int (S.M.B.); agbenue@who.int (E.A.A.); rhanda.adechina@who.int (R.M.A.); nyarkok@who.int (K.A.N.); 4 INSSA, Université Nazi Boni de Bobo-Dioulasso, Bobo-Dioulasso 1091, Burkina Faso; 5 The Discipline of Public Health Medicine, School of Nursing and Public Health Medicine, Howard College Campus, University of KwaZulu Natal, Durban 4041, South Africa

**Keywords:** vaccine safety, safety monitoring, pharmacovigilance, vaccine research and development, new vaccine introduction, manufacturer accountability, regional collaboration, Africa

## Abstract

The 8th meeting of the African Advisory Committee on Vaccine Safety (AACVS), constituted in 2021, convened by the Vaccine Research and Innovation Unit within the Vaccine Preventable Diseases Program, WHO Regional Office for Africa, was held virtually from 14 to 16 April 2025. The meeting brought together independent vaccine experts to provide advice to the Regional Director, WHO, on vaccine safety issues critical to the African region. Discussions focused on critical updates regarding ongoing regional outbreaks, safety data, and associated safety concerns, with emphasis on newly introduced vaccines, including the malaria vaccines (RTS, S and R21), the MenFive pentavalent meningitis vaccine, and the Mpox vaccines—MVA-BN and LC16—alongside the ongoing Mpox response. The Committee conducted a deep dive into comprehensive safety considerations for new vaccine introduction, active surveillance strategies, strengthening the responsiveness of pharmacovigilance systems, and advancing vaccine research and development in Africa. Key observations highlighted significant gaps in safety surveillance systems. These included delays in data collection, access, and signal detection; a lack of harmonized real-time monitoring frameworks; the underutilization of digital technologies; and inadequate manufacturer responsibilities and accountability in post-market safety monitoring. The meeting concluded with a call to action emphasizing the need for sustainable pharmacovigilance funding mechanisms, improved regional coordination, real-time data sharing, standardized early safety study protocols, strengthened manufacturer accountability, and investments in risk communication and community engagement to bolster public trust. Strengthening vaccine safety systems and enhancing regional collaboration were recognized as urgent priorities to support the safe and effective deployment of vaccines and protect public health across Africa.

## 1. Introduction

Vaccines have been and continue to be among the most impactful tools in public health, saving millions of lives, preventing outbreaks, and strengthening health systems [1,2].

Yet, their success depends not only on their development but on the trust communities place in them, trust that is built on the assurance that vaccines are safe, of assured quality, effective, and monitored with rigor and transparency [3,4,5,6].

Vaccines are typically authorized following rigorous clinical trials that assess their safety, efficacy, and quality. However, some vaccines may be deployed under Emergency Use Authorization during outbreaks and public health emergencies, based on partial or evolving data [7,8,9]. In such cases, timely and continued monitoring and real-world data collection and sharing become essential to ensuring their safe and effective use [10].

Despite Africa accounting for 18% of the global population, it hosts less than 2.5% of global clinical trials. This reality highlights the need for stronger safety monitoring systems to ensure that vaccines used on the continent are supported by data that reflect African populations and settings [11].

The 8th Meeting of the African Advisory Committee on Vaccine Safety (AACVS), organized by the WHO Regional Office for Africa’s AVAREF Secretariat within the Vaccine Research and Innovation Unit (VRIU) on 14–16 April 2025, brought together independent experts and stakeholders to discuss vaccine safety issues in the African Region. The AACVS provides independent, evidence-based advice to WHO AFRO, ensuring vaccine safety remains a priority and that immunization efforts are guided by sound scientific and technical guidance. The AACVS plays a critical role in shaping robust vaccine safety policies and practices that impact millions of lives. Since the AACVS’ establishment in 2021, the Committee has provided vital expert guidance on vaccine safety and the reporting and assessment of adverse events and has supported decision making during emergency vaccine rollouts.

The purpose of the 8th AACVS meeting was to review vaccine safety surveillance in the African Region, focusing on Mpox vaccines; newly introduced vaccines like RTS, S, R21, and MenFive; and those under Emergency Use Authorization. In the current Mpox response, the validation of the Mpox vaccine’s safety profile has been hindered by the timely availability and lack of access to safety data, weak surveillance systems, and low levels of spontaneous reporting. These gaps have delayed signal detection and the rapid generation of the evidence needed for effective safety monitoring and regulatory response. Additionally, some safety signals observed following the use of the MenFive meningococcal meningitis vaccine in Africa were not reflected in the original product package inserts, underscoring the need for region-specific safety monitoring. The persistent lack of sustainable funding for safety surveillance and studies across the region continues to be a major barrier to improving pharmacovigilance capacity and practice.

The meeting aimed to strengthen post-authorization vaccine safety preparedness and surveillance strategies, address progress and challenges with adverse event reporting, address vaccine hesitancy, and support innovation in vaccine research.

## 2. Summary of the 3-Day Agenda

The three-day meeting agenda focused on reviewing updates on regional outbreaks, vaccine safety data, and associated safety issues with particular attention to Malaria vaccines (RTS, S/R21), the MenFive meningitis vaccine, and Mpox vaccines, including the ongoing Mpox response in Africa. The sessions also included an in-depth examination of comprehensive safety considerations and active surveillance strategies for new vaccine introduction and existing vaccines in Africa, strengthening the responsiveness of the vaccine pharmacovigilance (PV) systems across the continent, and advancing vaccine research and development initiatives in Africa.

## 3. Key Observations

New vaccine introduction in Africa requires robust, systematic safety monitoring due to limited clinical trial data from the region.

Timely and rapid data collection, analysis, and sharing are essential to safe and effective vaccine deployment, but current systems are slow and ineffective, as seen in the Mpox response.

There is a lack of a consistent, standardized, ready-to-deploy system for monitoring and assessing adverse events during the introduction of new vaccines, including those under emergency use.

The absence of regional model protocols and frameworks hampers the timely and standardized conduct of safety studies during new introduction and public health emergencies, with inadequate funding and resource allocation further limiting effective implementation.

Safety surveillance during new vaccine introduction is severely underfunded, with limited-to-no resources allocated for data collection, adverse event case management, or the deployment of trained personnel and digital tools, hindering progress in regional pharmacovigilance systems.

Real-time monitoring systems must be strengthened to improve efficient safety data collection during vaccine rollouts.

The early use of available vaccine safety data needs to be improved to better inform public health decision making.

Manufacturers’ responsibilities in safety monitoring need stronger enforcement, including safety profile updates on product information, accountability for adverse events, and the funding of safety studies.

Pharmacovigilance systems in Africa are fragmented and uncoordinated, delaying safety responses and thus limiting timely and effective data collection and analysis.

Safety monitoring remains reactive in many low-resource settings, resulting in delayed detection and response to emerging safety issues.

There is an absence of a harmonized, real-time regional safety surveillance framework and the underutilization of available digital technologies for safety monitoring.

The fragmentation of systems and the withholding of available safety data across the region limit transparency and coordinated action.

There is poor safety data sharing between vaccine manufacturers and national regulatory authorities (NRAs), Expanded Programme on Immunization (EPI), and PV systems, weakening the availability and quality of safety information. This highlights a critical gap in collaboration between the PV and vaccination programs needed to strengthen data collection and timely information exchange.

Widespread misinformation and negative narratives on social media are contributing to increased vaccine hesitancy, highlighting the need for strengthened and more effective communication strategies.

## 4. Call to Action

To ensure the continued success of vaccination programs and the safety monitoring and surveillance of the introduction of new vaccines (including those under EUA), improve the responsiveness of safety surveillance systems, and enhance vaccine development across the continent, we urge all stakeholders—governments, regulatory authorities, manufacturers, regional bodies, and partners—to commit to the following actions.

### 4.1. Secure Sustainable Funding for Pharmacovigilance

Advocate for institutionalized post-market surveillance funding mechanisms, with mandatory contributions by vaccine manufacturers.

Integrate pharmacovigilance funding into national vaccine deployment plans to guarantee long-term sustainability.

### 4.2. Enhance Manufacturer Responsibility and Accountability

Ensure manufacturers are actively engaged in post-marketing studies, safety monitoring through mandatory contributory funding to NRAs while remaining independent of vaccine safety studies within the region.

Require manufacturers to update safety profiles by using real-world African safety data and assume liability for serious adverse events following the use of their vaccines and their management.

Mandate manufacturers to enhance their contributions to post-market surveillance by ensuring they update product information based on emerging safety data specifically data from Africa, where such data may not have been available during clinical trials.

### 4.3. Strengthen National and Regional Safety Surveillance Systems

Promote independent, transparent, and regionally coordinated safety monitoring systems to enhance real-time data sharing and timely signal detection, as well as supporting transparency and rapid response across Member States, as seen with the Vaccine Monitoring Platform, a collaboration between the European Medicines Agency (EMA) and the European Centre for Disease Prevention and Control (ECDC) [12].

Strengthen formal mechanisms for timely and transparent safety data sharing among vaccine manufacturers, NRAs, EPI programs, and pharmacovigilance systems to improve the coordination, availability and use of safety data for decision making.

Invest in national pharmacovigilance systems by training personnel, enhancing AEFI reporting mechanisms, and securing sustainable funding as part of continuous and early preparedness plan in case of emergencies.

Expand technical support to NRAs and countries to strengthen Adverse Events Following Immunization (AEFI) detection, reporting, investigation, and causality assessment capacities.

Provide targeted technical support to NRAs, offering access to digital tools including those with artificial intelligence capabilities, training experts in PV safety data analysis, and contributing to regional safety platforms for a faster, more integrated response.

### 4.4. Advance Regional Coordination and Data Integration

Develop a harmonized continental pharmacovigilance strategy to facilitate timely access to safety data and reduce system fragmentation.

Build a continental data-sharing structure to enable collaborative, real-time safety data flow and coordinated responses to emerging safety concerns.

Bolster the role of VRUI (AVAREF Secretariat) in coordinating regulatory responses and updating vaccine safety profiles.

Strengthen collaboration among national regulatory authorities, manufacturers, public health organizations, academics, and key regional organizations to enhance coordinated oversight and improve efficiency.

### 4.5. Enhance Safety Surveillance

Implement adaptable, regionally coordinated safety surveillance frameworks and protocols to support the consistent and rapid collection of safety data and safety monitoring during new vaccine introduction. These would serve as emergency-ready vaccine safety infrastructure designed to be deployed as a comprehensive package during EUA and new vaccine introduction. This approach is a critical component of preparedness planning, enabling the timely generation of safety data. It mirrors established models such as the Safety monitoring toolkit developed by the of the European Medicines Agency and ready-to-deploy protocols published by the US FDA during the COVID-19 pandemic, each tailored to different safety outcomes. This strategy reflects key lessons learned from the COVID-19 pandemic and builds on best practices from both the EMA, US FDA, and manufacturers of vaccines like Pfizer.

Strengthen passive surveillance and build capacity for active surveillance through simplified cohort event monitoring, the establishment of sentinel hospitals, real-time data dashboards, and regional coordination platforms.

### 4.6. Leverage Digital Innovations

Harness digital transformation opportunities offered by electronic health records (EHRs), including leveraging the untapped wealth of data from public health programs.

Strengthen interoperability between EHR systems to facilitate real-time data sharing and analysis, ensuring seamless integration with broader health systems.

Employ advanced analytics, such as machine learning and artificial intelligence to identify safety signals, predict adverse events, and optimize decision-making processes for improved vaccine safety monitoring and responsiveness [13].

### 4.7. Invest in Risk Communication and Public Trust

Build strong proactive, transparent, and continuous data-driven risk communication strategies to counter misinformation, foster public trust, and support informed decision making during vaccine rollouts. These efforts should begin before vaccination campaigns commence. Communication should engage diverse stakeholders, including the communities, healthcare providers, patient groups, research institutions, and relevant actors, and be delivered across multiple platforms and channels.

Leverage advanced technologies such as artificial intelligence to monitor and analyse real-time misinformation trends across digital platforms; this would further promptly counter the vaccine hesitancy driven by social media misinformation [14].

Design communication efforts that are community-centred and culturally sensitive, aligned with scientific evidence, incorporating insights from local demographic and epidemiological data.

### 4.8. Prioritize High-Impact Vaccine Research and Development

Promote an African-led vaccine research agenda, aligned with disease burden, equity, and feasibility priorities, focusing on endemic diseases such as malaria and tuberculosis, while addressing emerging and re-emerging infectious diseases.

The AACVS should provide strategic support for vaccine research and development, identify regional R&D priorities, and actively collaborating with existing scientific networks and research consortia.

Strengthen information flow among African countries and stakeholders to promote a unified, coordinated approach to vaccine R&D.

## 5. Conclusions

The convening of the 8th AACVS meeting marked a pivotal moment, coming on the heels of major advancements such as the recent introduction of vaccines for Malaria (RTS, S and R21) and Mpox, along with the anticipated rollout of the MenFive pentavalent meningitis vaccine. The gathering presented a tremendous opportunity to reflect on the progress, review the evidence, share experiences, and strengthen the pharmacovigilance systems that ensure the safety of every dose administered. It was also critical to identify gaps, align strategies, and reinforce collaborations, all with the shared goal of protecting public health and maintaining the confidence of African communities as the region moves forward with future vaccine introduction.

By working together across sectors and borders with strong accountability, sustainable investment, and regional solidarity, we can build a resilient vaccine safety and innovation ecosystem that protects every community in Africa and beyond.

## Abbreviations

The following abbreviations are used in this manuscript:

AACVS	Africa Advisory Committee on Vaccine Safety
AVAREF	Africa Vaccine Regulatory Forum
EPI	Expanded Programme on Immunization
NRA	National Regulatory Authority
PV	Pharmacovigilance
VRIU	Vaccine Research and Innovation Unit (VRIU)
VPD	Vaccine Preventable Disease
WHO	World Health Organization
